# Implementation of the World Health Organization’s Package of Essential Noncommunicable Disease Interventions: an overview of reviews

**DOI:** 10.3389/phrs.2026.1609607

**Published:** 2026-09-18

**Authors:** Hongyi Xu, Yiming Hu, Maoshu Li, Jing Yang, João Joaquim Breda, Jing Wu, Alarcos Cieza

**Affiliations:** 1 Department of Noncommunicable Diseases, Rehabilitation and Disability, World Health Organization, Geneva, Switzerland; 2 National Center for Chronic and Noncommunicable Disease Control and Prevention, Chinese Center for Disease Control and Prevention and Chinese Academy of Preventive Medicine, Beijing, China; 3 Department of Quality of Care & Patient Safety, World Health Organization Regional Office for Europe, Athens, Greece

**Keywords:** integrated care, low-resource settings, noncommunicable diseases, primary healthcare, WHO PEN

## Abstract

**Objectives:**

To synthesize the evidence on the implementation of the World Health Organization’s Package of Essential Noncommunicable Disease Interventions (WHO PEN).

**Methods:**

This narrative overview of reviews draws on evidence from systematic reviews and other review articles.

**Results:**

The findings reveal substantial gaps and challenges in public primary health facilities across many low- and middle-income countries, particularly in workforce capacity and the availability of essential medicines, diagnostics, and equipment. Several delivery strategies—such as high-risk population screening, task-shifting to non-physician health workers, team-based care, integrated HIV-NCD services, and the use of community pharmacies—were shown to effectively support WHO PEN implementation and expand access to NCD services. Strengthening the organization and resourcing of healthcare teams across system levels is essential. Additional promising approaches include leveraging existing service platforms, reinforcing district hospitals, adopting more patient-centered and prevention-oriented models within traditionally hospital-centric systems, and improving quality of care.

**Conclusion:**

WHO PEN and similar strategies can improve NCD service delivery and outcomes, but sustained investments, context-specific adaptations, and longer-term evaluations are needed to overcome persistent implementation barriers.

## Introduction

### WHO’s package of essential noncommunicable disease interventions

The rising global burden of noncommunicable diseases (NCDs) poses a significant challenge to health systems worldwide, with a disproportionate impact on low- and middle-income countries (LMICs) [1]. In response, the World Health Organization developed the Package of Essential Noncommunicable Disease Interventions (WHO PEN). WHO PEN was considered a minimum standard to address NCDs in primary care in low-resource settings, strategically prioritizing prevention and strengthening early detection and timely treatment of common NCDs [2]. First published in 2010 and updated in 2017 and 2020, the WHO PEN initially focused on simplified clinical protocols for managing hypertension and diabetes, as well as interventions to prevent cardiovascular disease (CVD) using the total cardiovascular risk assessment [3]. The 2020 update expanded the package to include modules for managing hypertension, diabetes, asthma, and chronic obstructive pulmonary disease (COPD), along with guidance on early cancer diagnosis, lifestyle counseling, self-care, and palliative care.

More than 40 countries across all WHO regions have begun strengthening health systems and primary healthcare services for NCDs through the implementation of WHO PEN. WHO—through its regional and country offices, along with international experts from collaborating centers—has provided technical support to facilitate PEN implementation [4–6]. A stepwise approach is recommended for implementing WHO PEN, including advocacy and stakeholder engagement, identifying pilot or demonstration sites, assessing primary health facility capacity and NCD services, developing a service delivery package aligned with PEN, capacity building, evaluation and review, integration into health financing processes, addressing medicines and diagnostic bottlenecks, and planning for scale-up.

Effective implementation of WHO PEN is expected to strengthen national capacity to expand NCD services, reduce inequities in access to care, and improve support for people living with NCDs.

### Complex evidence of WHO PEN implementation

The translation of WHO PEN from global recommendations into national policy and frontline practice is complex and highly heterogeneous. Because the package comprises multifaceted interventions and countries differ widely in capacity, resources, and health system contexts, the scope and pace of implementation vary substantially across regions. For example, in the WHO African Region, adapting and operationalizing WHO PEN has been a priority for many countries [5]. In the WHO Eastern Mediterranean Region, NCD emergency service kits—adapted from WHO PEN—have been developed to support NCD care in humanitarian settings [7]. In the WHO European Region, WHO PEN has been used to strengthen the quality of NCD care at health clinics [8].

When WHO PEN was first released, evidence on several core components—such as task-shifting for cardiovascular disease management and population-based screening—remained limited [9–11]. Since then, research has expanded, and new findings continue to emerge. However, despite the growing body of evidence on WHO PEN implementation—including facility readiness, service delivery, and health outcomes—the findings remain fragmented and difficult to interpret collectively.

This fragmentation underscores the need for a review approach capable of accommodating heterogeneity and synthesizing disparate findings. A comprehensive synthesis is essential to consolidate lessons learned, identify common challenges, and inform the refinement and optimization of the PEN protocol.

Consequently, this review systematically synthesizes evidence from existing reviews on WHO PEN implementation. Our aims are to identify the challenges and capacity gaps encountered during implementation, assess the effectiveness of various delivery and implementation strategies, and map the key barriers and facilitators influencing scale-up. Ultimately, this work seeks to strengthen the evidence base for WHO PEN and support the adoption of cost-effective strategies for preventing and controlling NCDs in low- and middle-income countries.

## Methods

### Review design

We conducted an overview of reviews with narrative synthesis. The protocol for the broader two-stage systematic review, of which the present overview constituted stage 1, was registered in PROSPERO (registration number: CRD420251064835) and published in the British Medical Journal Open in 2026 [12]. The review was conducted and reported in accordance with the Preferred Reporting Items for Overviews of Reviews (PRIOR) and relevant items of the PRISMA 2020 statement.

### Search strategy

We searched PubMed, Web of Science, Cochrane Library, Embase, and Google Scholar for reviews of WHO PEN implementation or WHO PEN-aligned interventions published from January 2010 to June 2025. Following pilot searches using broader combinations of synonyms and Medical Subject Headings, the final search was refined to the terms “package of essential noncommunicable” OR “WHO PEN”. The reference lists of eligible reviews were also searched to identify additional publications.

### Eligibility criteria

Consistent with stage 1 of the published protocol, we included systematic reviews, scoping reviews, and other explicitly described evidence reviews that examined WHO PEN implementation or evidence directly relevant to its implementation. Eligible reviews either [1] evaluated WHO PEN implementation, adaptation, outcomes, barriers and facilitators, or implementation capacity or [2] assessed a clearly defined WHO PEN-aligned component, such as cardiovascular risk assessment, task-sharing, integrated NCD care, access to essential medicines and diagnostics, or NCD service delivery through primary care or community platforms.

We excluded reviews that did not report implementation-related findings, lacked a clear link to WHO PEN or a defined WHO PEN component, or focused only on the clinical management of an individual condition.

In accordance with the published protocol, no geographical or language restrictions were applied during study identification. However, the primary synthesis focused on LMICs. We included reviews covering both high-income countries and LMICs, but separately reported LMIC findings that were used to support LMIC-specific conclusions.

## Results

Of the 22 reviews and systematic reviews screened, we selected 10 based on the inclusion criteria. An additional two reviews were identified through reference sourcing because they reported interventions aligned with WHO PEN. We included one preprint systematic review because it was highly relevant to the PEN-aligned strategy under investigation (Table 1). We employed a narrative, overview-of-reviews approach to synthesize the broad and heterogeneous evidence directly relevant to the WHO PEN and its implementation. The included reviews were analyzed and categorized into three thematic areas: health facility capacity (n = 4); implementation strategies (n = 5); and outcomes, barriers, and facilitators of WHO PEN implementation (n = 3) (Table 1).

**TABLE 1:** Summary of reviews on implementing the World Health Organization’s Package of Essential Noncommunicable Disease Interventions (WHO PEN) (Global, 2025).

Author	Relevance to WHO PEN implementation	Quality assessment	Location	Study characteristics	Review findings, focusing on LMICs	Conclusions
Health facility capacity to implement WHO PEN (n = 4)						
Albelbeisi [13]	Public sector capacity by WHO PEN standards (essential medicines, basic equipment, and diagnostic investigations)	Quality issues discussed included heterogeneity of results, study timing, sample sizes, and assessment at a single time point	Benin, Bhutan, Eritrea, Ghana, Nepal, Sri Lanka, Sudan, Suriname, Syria, Uganda, Vietnam, Zambia	Study design: Five original cross-sectional studies (2012–2018) reported public-sector capacity to implement NCD interventions in 12 countries. The sample included 304 public-sector facilities, of which 173 (56.9%) were PHC facilities	The readiness index scores for 33 essential medicines, 7 diagnostic investigations, and 13 basic equipment items ranged from 13.5% to 51%, 0.0%–59.4%, and 29.2%–51.2%, respectively	Critical gaps were identified in public-sector capacity to prevent and control NCDs in the 12 LMICs in terms of essential medicines, basic equipment, and diagnostic investigations. Policymakers should increase commitments
Kibirige [14]	Assess availability and affordability of essential diabetes medicines and diagnostic tests using WHO PEN standards	Newcastle–Ottawa scale quality assessment and funnel plots to assess publication bias	Benin, DR Congo, Cameroon, Ethiopia, Eritrea, Malawi, Mozambique, Nigeria, Senegal, Sudan, Tanzania, Uganda, Zambia	Study design: 21 cross-sectional studies (2000–2021) covering 2,215 health facilities (mostly public) across 15 African countries. Pooled availability of medicines and diagnostic tests. Meta-analysis with forest plots using a random-effects REML model	Availability was suboptimal for all five essential medicines and three diagnostic tests studied but was particularly poor for the HbA1c test and intermediate-acting insulin in Africa. Insulin, HbA1c tests, and lipid profile tests were costly	Given inequities in access and cost, pragmatic country-specific strategies are needed
Tesema [5]	Assess progress in implementing WHO PEN and integrating NCD services into PHC in Africa	Review. No quality assessment	All 47 Member States of the WHO African region	Based on the WHO Multi-country data review, which covered 47 countries, the assessment primarily encompassed four key areas: Policy and guidelines, PHC workforce, essential medicines and technologies, and information systems	No African country fully achieved all NCD integration targets; 7 countries met none. Only 30% had approved NCD guidelines, 13% ensured the availability of essential medicines, and 11% had core technologies in primary care No correlation was found between a country’s GDP *per capita* and target attainment, whereas a modestly negative correlation emerged with out-of-pocket expenditure	Progress in integrating NCD services into African primary care remains inadequate and uneven. To address this, essential NCD service integration at the PHC level requires intensified efforts across WHO AFRO Member States
Gupta [15]	First-referral-level hospital capacity (equipment and medication) to deliver NCD interventions (for four acute and nine chronic NCDs) in low-income countries	Not assessed. Results were reported as 95% CIs. The review included some discussion of missing data	Democratic Republic of Congo, Ethiopia, Haiti, Malawi, Nepal, Senegal, and Tanzania	Cross-sectional analysis of service provision survey assessments in 797 public first-referral-level hospitals in eight low-income countries	Compared to health centers, first-referral-level hospitals had better availability of commodities for acute epilepsy (40%–95%) and stage 1–2 hypertension (28%–83%). Overall, low availability of the minimum required equipment and medications to provide adequate diagnostic and therapeutic interventions for nine chronic conditions (including diabetes) and four acute presentations of chronic conditions at first-referral level hospitals	There is a need to prioritize the decentralization of NCD interventions to first-referral-level hospitals to increase care availability and promote more equitable access
Implementation strategies (n = 5)						
Davari [21]*	Cost-effectiveness analysis of screening the general population for hypertension, including an economic evaluation of the PEN program in Indonesia	Assessed. Six studies were excluded using predefined appraisal criteria for pharmacoeconomic studies. The quality of the included studies was generally low	Australia, Bhutan, Holland, Indonesia, Kenya, Nigeria, Thailand, USA, UK, Vietnam	Eleven studies published since 2000 (four included participants aged 40 years and older, three included participants aged 50 years and older, two included participants aged 30 years and older, and two included participants aged 15 years and older)	Cost-effective screening strategies: screening adults aged 55 years and older while increasing treatment coverage by 20% (Vietnam); screening high-risk groups aged 40 years and older (Indonesia, Thailand, Bhutan); screening adults aged 35 years and older integrated with community awareness (Kenya, urban slum); and screening and treating hypertension, with a wide range of uncertainty at a willingness-to-pay threshold of one GDP *per capita* per DALY averted (rural Nigeria) Barriers include limited access to healthcare, an inadequate health workforce, poor financial protection, and low health literacy. Integrating multiple interventions, task-shifting, and using local opportunities to reach target populations are important options for improving opportunistic screening	Screening high-risk populations aged 40 years and older is cost-effective, whereas screening general population is not. Health facility-based opportunistic screening alone is not sufficient. Expanding opportunistic screening to universal coverage for target populations is cost-effective
Muratalieva [8]	Strategies to address NCD care at the PHC level and systemic issues such as shortages of human resources and poor quality of care through PEN implementation	Scoping review. No quality assessment	Armenia, Azerbaijan, Belarus, Georgia, Kazakhstan, Kyrgyzstan, Moldova, Russia, Tajikistan, Turkmenistan, Ukraine and Uzbekistan. Central Asia and Eastern Europe	Study design: 96 studies (2010–2020) described hypertension and diabetes management and control and the role of PHC. Two countries piloted WHO PEN. Results were analyzed using the WHO Health system Building Blocks	NCD care at the PHC level continues to be provided predominantly by specialists, with a focus on treatment rather than preventive services Factors: Excessive workload, shortage of human resources, poor quality of care Low motivation of doctors and nurses to work more and cover health promotion and counseling on NCD-related risk factors Improve the quality of the PHC system through a patient-centered approach by strengthening the gatekeeping function and introducing counseling on NCD risk-factor prevention through PEN implementation. Population health outcomes are improved	WHO should take a more proactive position by promoting the adoption and implementation of PEN and offering best practices Countries should revise their priorities toward strong PHC systems with a patient- and prevention-centered approach
Joshi [16]	Evidence on task-shifting for CVD risk management and how to implement it	Review. No quality assessment	Argentina, Canada (Indigenous communities), Colombia, Ghana, India, Kenya, Malaysia, South Africa, Tanzania	Study design: Eight implementation case studies focusing on implementation challenges and facilitators for the prevention and management of CVD (types of tasks shifted, professional level, training, and challenges faced)	Both the potential and challenges of implementation of WHO PEN through task-shifting to NPHWs, including training needs, system-level barriers (inability to prescribe medicines, varying skill sets of NPHWs, high workload and staff turnover), and the importance of context-specific adaptation Tasks shifted included screening/monitoring, patient education, follow-up, adherence management, and triaging referrals and cases for management by physicians Field lessons: Hypertension training programs for NPHWs ranged from 3 to 7 days, with most including refresher training and on-the-job clinical decision support through training manuals or mHealth tools/platforms The review also emphasized the need to supervise and monitor implementation to ensure high-quality healthcare for the community	Task-shifting to non-physician health workers (NPHWs) is feasible and scalable for managing hypertension in low-resource settings
van der Mannen [17]	Integrated care: HIV and NCD integration in Sub-Saharan Africa (SSA) through PEN/HEARTS implementation	Review. No quality assessment	Kenya, Lesotho, Malawi, Namibia, Nigeria, South Africa, Tanzania, Uganda	Study design: 14 case studies (2010–2020) of the intersecting epidemics of HIV and NCDs and the integration of HIV and NCD services (asthma, cervical cancer, COPD, diabetes, epilepsy, hypertension, and mental health) Two countries piloted WHO PEN/HEARTS (hypertension and CVD risk). Most tasks were shifted to nurses or community health workers	Benefits of integrating HIV and NCD care: improved clinical outcomes and retention rates, stable HIV control, increased access to NCD services (screening, patient counseling, and medication), and cost-effectiveness Models mostly integrated NCD care into existing HIV care models, implemented HIV and NCD services simultaneously, or integrated HIV care into existing NCD care Barriers include the lack of guidelines and inadequate infrastructure	NCD and HIV healthcare integration in SSA is feasible, and models of service integration are related to the implementation context
Gentilini [18]	Delivery of NCD interventions included in WHO PEN via community retail pharmacies in LMICs	Assessed for RCTs and observational studies using Cochrane collaboration tools. The majority of RCTs had quality concerns; one RCT and five observational studies had a high risk of bias	Brazil, Egypt, Ghana, Indonesia, India, Iran, Malaysia, Nigeria, Pakistan, Puerto Rico, Thailand, among others	Twenty-three studies (1990–2022) (12 RCTs, 8 observational studies, 3 systematic reviews) (15 studies focused on diabetes, 5 on cardiovascular disease, 2 on cancer, and 1 on asthma). Three studies, conducted in Brazil and Pakistan, lasted 10–36 months	Positive outcomes included improvements in glycemic control and medication adherence for diabetes, blood pressure management and adherence for hypertension, and health behaviors for breast cancer, asthma, and cardiovascular disease risk management. Interventions were cost-effective for managing diabetes and hypertension in elderly patients Addressing resource constraints, legal barriers, and disease focus disparities is essential. Adequate training, financial incentives, and collaboration among stakeholders are crucial for integrating pharmacists into NCD care frameworks	Expanding NCD services through community pharmacies and pharmacists in LMICs shows potential for managing diabetes and hypertension in elderly patients Pharmacists can enhance education, screening, and management for NCDs, leading to better disease control and patient satisfaction
Early outcomes, barriers, and facilitators (n = 3)						
Tripathy [19]	Effectiveness, outcomes, and challenges of WHO PEN implementation	Not assessed. One included cost-effectiveness study used an estimated microsimulation model that lacked local data for its parameters	Bhutan, Gaza/Palestine, Kyrgyzstan, Indonesia, Jordan, Myanmar, People’s Republic of Korea, Philippines, Republic of Moldova, Samoa, and Tajikistan	Study design: 14 studies reported implementation outcomes up to 2020, excluding interventions that did not follow the WHO PEN protocol in primary care settings. A total of 10 countries were included	Nine recommendations should be addressed before implementation is scaled up A lack of essential medicines and manpower, poor recording and miscalculation of CVD risk scores, suboptimal recording of patient information, and loss to follow-up were the key challenges Outcomes: Effectiveness (clinical outcomes, prevalence reduction, and control of risk factors), patient perceptions, health system readiness, cost-effectiveness, adherence to the protocol, and performance/coverage	Targeted screening policy and expanding opportunistic screening may be more cost-effective Evidence on effectiveness was inconclusive, and long-term follow-up was lacking
Aminpour [4]	Barriers and facilitators of WHO PEN implementation	Assessed. One of 16 studies was excluded due to average or below-average quality. Thirteen included studies received a quality rating of “good.”	Bhutan, Gaza/Palestine, Ghana, Indonesia, Iran, Jordan, Kyrgyzstan, Moldova, Myanmar, Nepal, South Africa	Study design: 15 original studies published from 2014 to 2020 in 13 countries. The studies were in English and Persian and were mostly evaluations, including four qualitative and two mixed-methods studies	Barriers and facilitators were categorized into 2 main themes and 7 subthemes Organizational factors including human resources, service delivery, structure, leadership/governance, data and information, resources, and financing were identified as barriers and facilitators. Knowledge and culture, as well as economic and social factors, were identified as contextual factors	Policymakers and health system managers should consider these factors when implementing WHO PEN and similar packages
Gunawan [20]	Obstacles encountered in implementing WHO PEN	Review. No quality assessment	Uganda, Republic of Moldova, China, Palestine, Mexico, Bangladesh, Democratic Republic of the Congo, Ethiopia, Haiti, Malawi, Nepal, Senegal, Tanzania, Vietnam, Jordan, Nigeria, Syria, Indonesia	Study design: 17 original studies published from 2002 to 2022, in English, predominantly evaluation studies, including 12 quantitative, 3 qualitative, and 2 mixed-methods studies	The implementation of minimum service standards for NCDs at the PHC level faces six main barriers: 1) standardized constraints and policy objectives; 2) resource constraints; 3) constraints related to the characteristics of implementing organizations; 4) communication barriers between organizations; 5) constraints associated with implementers’ attitudes; 6) socio-environmental constraints within the community	Governments and health authorities should adjust unrealistic policy targets, ensure resource availability, and strengthen inter-organizational coordination to effectively implement policies for NCDs

Quality appraisal refers to the assessment reported by the original evidence source. “No quality assessment” indicates that the source did not describe the use of a structured appraisal or risk-of-bias tool.

*This systematic review was available as a preprint at the time of our review. We included this study because it was highly relevant to the PEN-aligned strategy under investigation.

### Effectiveness of WHO PEN and PEN-aligned implementation strategies

Because WHO PEN is primarily designed for LMIC contexts, this synthesis prioritizes LMIC-specific findings. For reviews that included both HICs and LMICs, LMIC-specific findings were extracted where available. Where the original reviews did not disaggregate findings by country income group, we report the evidence as mixed-income evidence and interpret it cautiously.

Evidence on the effectiveness of WHO PEN implementation in LMICs was limited, but the available studies suggest potential benefits in selected settings. The systematic review by Tripathy et al. [19] examined studies from countries including North Korea, Bhutan, Myanmar, Moldova, Gaza/Palestine, and Indonesia and reported improvements across several domains. Studies from North Korea, Bhutan, and Myanmar showed reductions in population-level cardiovascular risk factors, including blood pressure, blood glucose, and smoking rates. Evidence from Moldova suggested improved treatment control and medication adherence among patients with hypertension and diabetes. Although a long-term follow-up study in the Gaza Strip did not find statistically significant improvements in clinical indicators, patients reported better quality of care and improved medication adherence after PEN implementation. Economic evaluations from Indonesia and Bhutan also suggested that targeted screening of high-risk individuals aged 40 years and older was more cost-effective than no screening or current practice.

Evidence from PEN-aligned strategies was also suggestive of benefit, although uneven. One systematic review on population-based hypertension screening [21] included evidence from both HICs and LMICs. Where LMIC-specific findings were available, the evidence supported targeted screening of high-risk adults aged 40 years and older rather than universal screening of all adults.

Task-shifting and team-based care were another important implementation approach. Joshi et al. [16] synthesized eight implementation case studies conducted across nine country settings, predominantly LMICs. Non-physician health workers (NPHWs) supported screening, patient education, referral, follow-up, and medication-adherence activities. In some settings, their responsibilities extended to hypertension diagnosis, medication initiation, and dose titration. Several interventions used mobile applications and clinical decision-support tools to assist NPHWs in screening, managing, and referring patients. The review suggested that task-shifting may expand cardiovascular risk management in settings with limited physician availability, provided that workers receive clearly defined responsibilities, structured training, supervision, and referral support.

Other service delivery models also showed promise, but the strength of evidence varied by setting. Van der Mannen et al. [17] reviewed integrated HIV-NCD service models and found that several LMICs, including Nigeria, Uganda, and Tanzania, had piloted WHO PEN or HEARTS for hypertension and CVD risk management within HIV services. These models used existing HIV platforms to provide NCD screening, counselling, referral, and chronic care follow-up. The findings indicated that integration was feasible in selected settings and boosts the sustainability of the HIV response while advancing universal health coverage and improving access to NCD care.

Gentilini et al. [18] systematically reviewed 23 studies of community retail pharmacy interventions in LMICs. Some studies reported improvements in glycemic control, medication adherence, blood pressure management, and health-related behaviors. However, the interventions, study designs, and outcomes were heterogeneous, and several randomized and observational studies had a high risk of bias. The findings therefore support the potential contribution of community pharmacies but do not establish their uniform effectiveness across LMIC settings.

Overall, WHO PEN implementation was associated with improvements in selected clinical outcomes, service delivery, treatment adherence, and patient-reported outcomes in some settings. Evidence for PEN-aligned strategies also points to potential benefits of targeted high-risk screening, structured task-shifting, integrated chronic care platforms, and pharmacy-based support. However, much of this evidence remains heterogeneous, and some findings are derived from mixed-income reviews that did not consistently disaggregate LMIC results. These findings should therefore be interpreted cautiously and should not be taken as evidence of full-scale PEN effectiveness across health systems.

### Challenges and capacity gaps in implementation

Despite evidence of potential benefit, the reviews consistently showed that many health systems lacked the basic capacity required to implement WHO PEN at scale. The main gaps concerned two related but distinct areas: access to essential medicines and diagnostics, including their availability and affordability; and broader PHC capacity, including workforce, clinical guidance, risk stratification, referral arrangements, and service organization.

### Availability and affordability of essential medicines and diagnostics

Facility readiness was commonly assessed using WHO PEN benchmarks for essential medicines, diagnostic investigations, and basic equipment. Albelbeisi et al. [13] synthesized five cross-sectional studies conducted between 2012 and 2018, covering 304 public health facilities across twelve LMICs, and found substantial gaps in service availability and readiness. Readiness scores varied widely across countries, ranging from 13.5% to 51% for essential medicines, 0.0%–59.4% for diagnostic investigations, and 29.2%–51.2% for basic equipment. No country reached the global benchmark of 80%.

Similar gaps were reported in Africa. Tesema et al. [5] assessed NCD service capacity across 47 African countries and found that only Cabo Verde and Mauritius reported having all ten essential NCD medicines and all six essential NCD technologies. Kibirige et al. [14] also reported suboptimal availability of five essential diabetes medicines and three diagnostic tests across 15 African countries, with particularly limited availability of intermediate-acting insulin and glycated hemoglobin testing.

Affordability represented a separate implementation challenge and patient-level barrier. Kibirige et al. [14] reported that the cost of short-acting insulin was equivalent to approximately 7 days’ wages, while glycated hemoglobin (HbA1c) testing and lipid-profile testing cost the equivalence of 8.6–12.6 days’ wages and 3.6–7.5 days’ wages, respectively. Broader financing and supply-chain constraints further affected continuity of access to medicines and diagnostics [4, 17], while weak financial protection was identified as an additional barrier in resource-constrained settings [21].

### Workforce capacity and service organization

The reviews also identified substantial gaps in workforce capacity, simplified clinical guidance, cardiovascular risk stratification, and the organization of long-term NCD care. Tesema et al. [5] found limited availability of simplified NCD guidelines and risk-based care at the PHC level. Only 14 countries had PHC guidelines for NCD management, and only two countries reported CVD risk stratification in more than 50% of PHC facilities.

Gunawan et al. [20] concluded that community health centers often failed to meet minimum NCD service standards because of insufficient human resources for NCD-related activities, including prevention, treatment, and risk factor control. Muratalieva et al. [8] further showed that, in several countries of the Commonwealth of Independent States, NCD care remained largely specialist- and hospital-based, with limited emphasis on prevention, continuity of care, and patient-centered PHC.

Gupta et al. [15] assessed service readiness in 797 public first-referral hospitals across eight low-income countries and found relatively better availability of commodities for acute epilepsy and stage 1–2 hypertension in first-referral hospitals across eight low-income countries. However, availability of medicines and equipment for diabetes remained limited, suggesting that referral-level facilities may help expand access for some conditions but cannot fully compensate for weak PHC capacity.

### Barriers and facilitators affecting implementation and scale-up

Barriers to WHO PEN implementation operated at patient, provider, organizational, and health-system levels. Aminpour et al. [4] classified barriers and facilitators into organizational factors and external social, cultural, and economic factors. Organizational factors included human resources, service delivery, health system structure, leadership and governance, information systems, and resources and financing.

In addition to the structural shortages described above, Tripathy et al. [19] highlighted implementation-process challenges including weak electronic registration systems, poor adherence to medication and lifestyle advice, loss to follow-up, limited home visits, weak referral linkages, lengthy reporting processes, limited community-based health education, and errors in calculating or recording CVD risk scores [19].

Service-delivery models also faced context-specific barriers. Integrated HIV-NCD models faced barriers such as unclear clinical guidelines, infrastructure limitations, supply chain weaknesses, privacy concerns, and patient flow challenges [17]. Community pharmacy-based models were affected by resource limitations, insufficient training, regulatory restrictions, unclear professional responsibilities, and weak referral linkages [18].

At the same time, the reviews identified several facilitators. These included structured training, refresher sessions, simplified treatment protocols, clinical decision-support tools, physician supervision, mHealth platforms, digital patient records, staff and patient education, stakeholder collaboration, reliable supply chains, financial protection, supportive policy environments, and stronger community engagement [4, 16–19, 21].

In practice, implementation is more likely to succeed when health workers are supported through training and supervision, referral and information systems function effectively, communities are engaged, and policy and regulatory environments enable NPHWs and community-based platforms to contribute safely to NCD care.

### Promising strategies for scale-up and contextual adaptation

Across the reviews, several strategies emerged as promising for scaling up WHO PEN in LMICs, although the strength and transferability of the supporting evidence varied. In LMIC settings where evidence was available, targeted screening of high-risk adults aged 40 years and older appeared more feasible and potentially more cost-effective than universal adult screening, particularly in resource-constrained settings [19, 21]. This approach requires simple risk-stratification tools, trained frontline personnel, and clear pathways for diagnosis, treatment, and referral.

Task-shifting and team-based care may expand access to screening, follow-up, lifestyle counselling, and risk-factor management, provided that frontline workers receive adequate training, supervision, and decision support [16]. These models require clearly defined scopes of practice, standardized protocols, competency-based training, ongoing supervision, decision-support tools, and effective referral arrangements.

Leveraging established service platforms was another recurring strategy. In settings with established HIV service infrastructure, integrating NCD screening, counselling, treatment, and follow-up into existing HIV care structures was reported as feasible and may extend access without creating entirely separate delivery systems [17]. Community retail pharmacies may also serve as accessible platforms for medication adherence support, blood pressure monitoring, diabetes care, and health education [18]. The applicability of these models therefore depends on local workforce capacity, professional regulation, training, financing, and referral linkages.

Evidence from first-referral hospitals suggested that these facilities may have better availability of selected medicines and equipment than primary health centers [15]. They may therefore have a complementary role in referral, supervision, and the management of selected acute or complex NCD conditions.

WHO PEN protocols themselves can be used as tools for PHC quality improvement. Reviews from countries of the Commonwealth of Independent States showed that PEN implementation encouraged more patient-centered care, stronger gatekeeping, counselling on risk-factor prevention, and auditing of clinical practices such as CVD risk assessment and statin prescription [8, 19].

However, these strategies should not be viewed as stand-alone solutions. Their success depends on the basic conditions repeatedly identified across the reviews: affordable medicines and diagnostics, adequate workforce capacity, reliable supply chains, simplified guidelines, information systems, and sustained financing [4, 14, 19, 20].

Table 2 summarizes the context-specific implementation considerations derived from these findings and indicates the scope and limitations of the evidence supporting each consideration.

**TABLE 2:** Review-derived implications for context-specific implementation of the World Health Organization’s Package of Essential Noncommunicable Disease Interventions (WHO PEN) in low- and middle- income countries (LMICs) (multiple LMICs, 2025).

Synthesis area	WHO PEN guidance	Key findings from included reviews	Context-specific implementation implications	Evidence basis
Primary healthcare readiness	Assess PHC infrastructure, workforce, equipment, diagnostics, medicines, and existing NCD services before implementation [2]	Reviews reported major gaps in medicines, diagnostics, equipment, workforce capacity, simplified guidelines, and cardiovascular risk assessment [5, 13–15, 20]	Scale-up should be preceded or accompanied by PHC readiness assessment and investment in minimum service inputs. May prioritize first-referral level hospitals where readiness is higher for implementation	Supported by several readiness-focused evidence sources, although indicators, settings, and data periods varied
Availability and affordability	Ensure access to essential medicines and technologies required for NCD management [2]	Availability did not ensure access. Medicines and diagnostic tests were frequently unaffordable, with additional financing and supply constraints [4, 14, 19, 20]	Monitoring should include availability, affordability, stock-outs, out-of-pocket expenditure, and continuity of access	Evidence was consistent in direction but concentrated in selected diseases and regions
Targeted screening and risk stratification	Use simplified cardiovascular risk assessment and management protocols [2]	Economic evidence suggested that screening adults aged ≥40 years or those at high cardiovascular risk may be more cost-effective than universal adult screening [19, 21]	Resource-constrained settings may consider targeted high-risk screening supported by simple risk tools and clear referral pathways	Mainly based on one mixed-income economic review (reprint) and one WHO PEN implementation review; transferability is context-dependent
Task-shifting and team-based care	Train PHC workers to assess, manage, and refer patients, with periodic supervision [2]	NPHWs supported screening, counselling, follow-up, adherence management, and selected risk-management tasks [16]	Task-shifting should include clear roles, standardized protocols, competency-based training, supervision, decision support, and referral arrangements	Based mainly on implementation case studies, comparative effectiveness evidence was limited
Integration with existing platforms	Integrate NCD care within PHC and existing service-delivery systems [2]	HIV/chronic-care platforms and community pharmacies showed potential to support NCD screening, counselling, treatment, follow-up, and adherence [17, 18] Prioritize decentralization NCDs interventions at first-referral level hospitals for broader range of NCDs	These models may be considered where infrastructure, regulation, workforce, financing, and referral systems are adequate	Each model was mainly supported by one topic-specific review; interventions and outcomes were heterogeneous
Quality and implementation fidelity	Use clinical records, audit tools, supervision, and agreed monitoring indicators [2]	Reviews identified inconsistent protocol adherence, weak follow-up, poor referral, errors in cardiovascular risk assessment, and variable patient experience [8, 19]	Implementation evaluation should assess whether WHO PEN is delivered as intended through audit, patient registers, referral tracking, and patient-experience measures	Supported by a small number of reviews; formal quality appraisal was absent in some sources

## Discussion

Although direct review-level evidence on WHO PEN implementation remains limited, that which is available suggests potential benefits in selected settings, including improvements in cardiovascular risk factors, treatment control, medication adherence, service delivery, and patient-perceived quality of care. Significant gaps and challenges persist in public primary health facilities in many LMICs, particularly in relation to health workforce capacity, availability and affordability of essential medicines, diagnostics and basic equipment, and the organization of long-term chronic care. Several PEN-aligned strategies, including targeted screening, task-shifting, integrated chronic care models, and community pharmacy-based support, appear promising, although the strength and transferability of the evidence vary across settings. Taken together, these findings support context-sensitive adaptation of WHO PEN rather than uniform implementation across health systems.

### Interpretation and implementation implications

A central finding of this review is that the availability of WHO PEN protocols alone is insufficient for successful implementation. Reviews of facility readiness documented major gaps in essential medicines, diagnostic investigations, and basic equipment. In one review based on facility assessments conducted between 2012 and 2018, none of the country-level mean readiness indices reached the 80% threshold applied by the review [13]. Although these historical estimates may not reflect current facility capacity, they demonstrate the scale of the documented readiness gaps. Other reviews also reported limited use of simplified NCD guidelines, weak cardiovascular risk stratification, shortages of trained staff, and continuing reliance on specialist- or hospital-centered models of NCD care [5, 8, 20]. In addition, a broader review of asthma care in India highlighted how limited diagnostic capacity, poor recognition of symptoms, and inadequate adaptation of clinical guidance may create additional implementation challenges in rural settings [22]. These capacity gaps help explain why implementation remains difficult even when policy commitment exists. They also demonstrate that implementation difficulties reflect broader weaknesses in PHC systems rather than isolated failures in program delivery.

Implementation barriers were not confined to service availability but affected the full care pathway from screening and diagnosis to treatment initiation, follow-up, and adherence. Affordability was a particularly important constraint, with evidence showing that essential diabetes medicines and diagnostic tests may remain financially inaccessible to patients even when available in facilities [14]. Weak supply chains, inadequate financing, insufficient training, poor referral linkages, limited home visits, weak information systems, and errors in CVD risk assessment were also reported [4, 19]. These health-system barriers may interact with patient-level factors. A scoping review cited for contextual interpretation, but not included in the formal synthesis, found that limited knowledge of disease and treatment, negative attitudes towards long-term medication, beliefs about medicines, and quality-of-life concerns contributed to poor medication adherence in LMICs [23]. These barriers suggest that sustainable implementation of WHO PEN is more likely to require broader primary care strengthening, including reliable procurement systems, simplified clinical tools, patient registration, referral tracking, supervision, and mechanisms for financial protection.

This overview of reviews and other similar studies [24, 25] identifies more practical and context-responsive approaches to adopting and implementing WHO PEN. Urban refugee, rural, and hospital settings, among others, all require more tailored approaches to implementing WHO PEN while meeting special needs or overcoming challenges, such as the need for dialysis services to save the lives of patients with chronic kidney disease, training health workers in rural facilities to recognize syndromes for the early diagnosis or referral of patients with asthma, and requiring specialists and doctors to adopt preventive interventions and support team-based care led by non-physician health workers.

Measurements of WHO PEN implementation require multidimensional data, ranging from service readiness and coverage to process indicators, quality-of-care assessments, risk-factor control, and clinical indicators.

Several delivery strategies may help translate WHO PEN recommendations into practice in resource-constrained settings. In LMIC contexts, targeted screening of high-risk adults, particularly those aged 40 years and older, appears more feasible and potentially more cost-effective than universal adult screening where resources are limited [19, 21]. This finding remains context-dependent, and its transferability is likely to vary according to the burden of local disease, screening costs, workforce capacity, and available referral and treatment services.

Task-shifting and team-based care may expand access to screening, lifestyle counseling, follow-up, and risk-factor management, especially when NPHWs are supported by clear protocols, training, supervision, and decision-support tools [16]. Integration of NCD care into established HIV or chronic care platforms may also be useful in settings with mature HIV chronic care infrastructure, although this evidence was largely derived from implementation case studies rather than comparative evaluations [17]. Additional studies similarly reported potential benefits from integrating HIV and NCD services, including greater service accessibility and continuity of chronic care, while also identifying substantial heterogeneity in integration models, target conditions, and reported outcomes [26, 27]. These contextual findings reinforce the need to adapt integration models to the organization and capacity of local services.

Community pharmacies may provide additional opportunities for blood pressure monitoring, medication adherence support, diabetes care, and health education, although their contribution depends on regulatory frameworks, training, referral linkages and collaboration with primary care providers [18]. A Ghanaian empirical study, cited as contextual evidence rather than as part of the formal synthesis, illustrated how unclear professional regulation may restrict community pharmacists’ involvement in hypertension and chronic disease management [28]. This example supports the broader finding that expansion of pharmacy-based care requires clearly defined professional responsibilities and appropriate regulatory support.

The level of confidence in these implementation strategies varied according to the supporting evidence. Findings on gaps in PHC readiness were reported consistently across several reviews, whereas task-shifting, service integration, and community pharmacy-based care were often supported mainly by single topic-specific reviews with heterogeneous underlying studies. These strategies should therefore be considered context-dependent, evidence-informed options rather than definitive or consensus recommendations.

Across these strategies, several common enabling conditions were evident: adequate workforce capacity, clearly defined professional roles, reliable supplies, functioning referral systems, supportive regulation, and sustained financing. Expansion of task-sharing, integrated care, or community-based delivery should therefore be based on local assessments of health-system capacity rather than on the assumption that a single delivery model will be transferable across all LMIC settings. Healthcare professionals, community health workers, pharmacists, patients, and carers may have complementary roles, but these roles should be defined according to local service organization, competencies, and regulatory frameworks.

The organization and equipping of healthcare teams across health system levels is another important consideration. In some settings, NCD care remains specialist-led and hospital-centered, with limited emphasis on prevention, continuity of care, and patient-centered primary care. At the same time, first-referral hospitals may have better availability of selected commodities than primary health centers [15]. This suggests that district hospitals, primary care facilities, community health workers, pharmacies, carers, and patients may all have complementary roles in WHO PEN implementation. Strengthening the role of first-level district hospitals and community-based actors may support decentralization and continuity of care.

Quality of care also requires more explicit attention. Some evidence suggests that PEN implementation can encourage more structured CVD risk assessment, counselling, clinical audit, and patient-centered care [8]. Nevertheless, process-related barriers such as loss to follow-up, poor adherence, weak referral linkages, and errors in recording risk scores indicate that implementation fidelity cannot be assumed [19]. Routine monitoring through standard process indicators, clinical audits, program reviews, and implementation evaluations should assess not only whether WHO PEN has been adopted but also whether it is delivered as intended and improves continuity, equity, and quality of care.

The evidence also points to areas where WHO PEN may require further refinement. Updates to its evidence base, norms, and standards could include clearer guidance on essential diagnostics and equipment, simplified tools for cardiovascular risk assessment in frontline practice, and practical examples of adaptation across different health system contexts. WHO and international partners could support implementation by disseminating lessons from successful PEN and PEN-aligned models, particularly those addressing affordability, workforce constraints, service integration, and quality improvement.

### Strengths and limitations

This overview brings together evidence on both direct WHO PEN implementation and interventions aligned with defined WHO PEN components. Its principal strength is the synthesis of otherwise fragmented evidence across facility readiness, implementation outcomes, affordability, workforce models, service integration, and implementation barriers. The LMIC-specific focus and the cautious treatment of mixed-income reviews also increase the relevance of the findings to the settings for which WHO PEN was primarily developed.

Several limitations should nevertheless be considered. First, the number of eligible reviews was small, and the included reviews differed considerably in their objectives, designs, settings, interventions, and outcome measures. This heterogeneity precluded quantitative pooling and required a narrative synthesis. Second, the methodological quality of the included reviews varied, and several reviews did not formally assess the quality of their underlying studies. Third, some mixed-income reviews did not report LMIC findings separately, limiting their contribution to LMIC-specific conclusions.

In addition, although no language restrictions were applied during study identification, the identified review-level evidence was predominantly published in English, and evidence from Latin America and Francophone Africa was limited. Consequently, implementation experiences reported in local-language publications, government documents, or regional literature may be under-represented, and the geographical representativeness of the synthesis may be incomplete.

### Future research

Future research should move beyond facility surveys, feasibility studies, and small pilot projects. More rigorous implementation research, longitudinal studies, pragmatic trials, and economic evaluations are needed to assess effectiveness, cost-effectiveness, equity, affordability, and sustainability across different LMIC contexts. Because follow-up periods were inconsistently defined and reported across the included reviews, future studies should report exact follow-up durations and distinguish early changes in service processes or patient experience from sustained clinical and population-level outcomes. Future research should also examine how workforce models, regulatory arrangements, financing mechanisms, and service-integration strategies interact with local health-system capacity. Future reviews should also disaggregate findings by country income group, region, health-system setting, and delivery model to generate more nuanced guidance for WHO PEN scale-up and adaptation.

### Conclusion

This overview of reviews found that WHO PEN and PEN-aligned strategies may support improvements in selected NCD service-delivery and patient outcomes in LMICs, including risk-factor control, treatment adherence, perceived quality of care, and access to chronic disease management. However, the evidence base remains limited, heterogeneous, and uneven in methodological quality.

Promising strategies include targeted high-risk screening, task-shifting and team-based care, integration of NCD services into existing chronic care platforms, and community pharmacy-based support. These strategies should be adapted to local context and implemented alongside investments in PHC readiness, affordability, supply chains, supervision, referral systems, and quality monitoring. Given the limitations of the available review-level evidence, conclusions about long-term effectiveness and health-system impact should be interpreted cautiously. More rigorous, context-specific, and long-term evaluations are needed to guide WHO PEN scale-up in LMICs.
